# Endoscopic Management of Obstructive Chronic Pancreatitis in a Dual Solid Organ Transplant Recipient: A Case Report

**DOI:** 10.7759/cureus.114931

**Published:** 2026-08-21

**Authors:** Muthafar I Alsufi, Muaath I Alsufi, Anas Zaher, Bashar J Qumseya

**Affiliations:** 1 Faculty of Medicine, University of Jordan, Amman, JOR; 2 Division of Gastroenterology, Hepatology and Nutrition, University of Florida, Gainesville, USA

**Keywords:** biliary stricture, chronic pancreatitis, ercp, exocrine pancreatic insufficiency, immunosuppression, kidney transplant, liver transplant, pancreatic stenting, type 3c diabetes

## Abstract

A 47-year-old woman with alcohol-related cirrhosis underwent simultaneous donor orthotopic liver transplantation and kidney transplantation. Her post-transplant course was complicated by a biliary anastomotic stricture, endoscopic retrograde cholangiopancreatography (ERCP)-related biliary perforation, and bile leak requiring percutaneous transhepatic cholangioplasty. Five years post-transplant, she developed progressive obstructive chronic pancreatitis with main pancreatic duct dilation to 10 mm, intraductal calculi, exocrine pancreatic insufficiency, and type 3c (pancreatogenic) diabetes with peak glycated hemoglobin of 9.6%. She underwent five serial ERCP procedures with pancreatic sphincterotomy, balloon dilation, stone extraction, and progressive plastic stent placement (5 Fr to 7 Fr), achieving modest weight gain, reduced pain, improved oral intake, and better glycemic control. Surgical decompression was deferred due to prohibitive operative risk. At nine months, the pancreatic stent was electively removed, and the patient remains under active multidisciplinary surveillance. This case demonstrates that serial endoscopic pancreatic duct decompression is feasible for managing obstructive chronic pancreatitis in dual solid organ transplant recipients when surgery carries excessive risk.

## Introduction

Orthotopic liver transplantation (OLT) is associated with significant biliary complications; approximately 6-12% of recipients develop biliary anastomotic strictures (BAS), a leading cause of graft dysfunction and retransplantation in the absence of timely intervention [1]. BAS arises primarily at the site of choledochocholedochostomy due to a multifactorial etiology that includes technical factors such as duct diameter mismatch and the number of anastomosed ducts, local ischemia, fibrosis, and the post-transplant immune response, and its management has evolved considerably over the past two decades [2]. Endoscopic retrograde cholangiopancreatography (ERCP) with balloon dilation and plastic stent placement has become the preferred first-line treatment for BAS, yielding stricture resolution rates of 70-94% with serial intervention. However, a subset of patients require percutaneous transhepatic cholangioplasty (PTC) or surgical revision when endoscopic access fails or complications such as bile leak and biliary perforation arise [3,4].

Repeated ERCP and papillary instrumentation are recognized risk factors for pancreatic injury, and patients who develop biliary complications post-OLT may therefore be at elevated risk for subsequent pancreatic ductal disease, though this relationship has not been systematically quantified. Chronic pancreatitis is a progressive inflammatory condition characterized by irreversible parenchymal fibrosis, leading to abdominal pain, weight loss, and exocrine and endocrine insufficiency. This endocrine failure often manifests as type 3c (pancreatogenic) diabetes, which is distinct from common type 1 or type 2 diabetes. It results from the widespread destruction of pancreatic parenchyma and carries a uniquely high risk of unpredictable hypoglycemia. When intraductal calculi and strictures cause obstructive physiology, treatment selection is guided by stone location, size, and number, with options including ERCP with sphincterotomy and stent placement, extracorporeal shock wave lithotripsy (ESWL), and surgical decompression or resection [5,6]. However, surgical interventions (such as the Puestow procedure) often carry a prohibitive risk in simultaneous liver-kidney transplant recipients. The compounding factors of heavy immunosuppression, severe malnutrition, and the absolute necessity of preserving dual allograft function make endoscopic management a vital, less invasive alternative.

Immunosuppressive therapy itself may also contribute to pancreatic dysfunction after transplantation. Tacrolimus, the calcineurin inhibitor used in most maintenance regimens, has direct toxic effects on pancreatic β-cells, reducing insulin secretion and insulin gene expression and promoting β-cell injury over time [7]. These effects, combined with the insulin resistance caused by corticosteroids, are well recognized contributors to post-transplantation diabetes mellitus (PTDM) [8]. In contrast, the effect of these drugs on exocrine pancreatic function is far less studied, and it is unclear whether chronic immunosuppression might also worsen ductal or parenchymal injury in patients who already have chronic pancreatitis.

To our knowledge, no prior case has been reported describing the management of progressive obstructive chronic pancreatitis in a recipient of simultaneous liver and kidney transplantation with prior ERCP-related biliary perforation and type 3c (pancreatogenic) diabetes. We present such a case, detailing the clinical course, endoscopic management, metabolic consequences, and multidisciplinary approach, with the aim of providing guidance for clinicians encountering similar high-complexity patients.

## Case presentation

A 47-year-old woman presented with a one-year history of chronic epigastric pain and a 30-pound unintentional weight loss over nine months. Her medical history was significant for alcohol-related cirrhosis secondary to long-standing alcohol use disorder (since discontinued) and alcohol-induced chronic pancreatitis predating her transplant. At the time of transplantation, she had no pre-existing diabetes, indicating preserved baseline endocrine function. Five years prior, she had undergone deceased donor OLT, followed two days later by deceased donor kidney transplantation. Her maintenance immunosuppressive regimen consisted of tacrolimus 4 mg twice daily, mycophenolate mofetil 500 mg twice daily, and prednisone 5 mg once daily. Her post-transplant course was complicated by a BAS, initially managed with ERCP, which was in turn complicated by biliary perforation and bile leak necessitating PTC with serial cholangioplasty, ultimately achieving complete stricture resolution. These severe biliary complications likely exacerbated the natural progression of her underlying pancreatitis, culminating in severe pancreatic atrophy, intraductal stones, and new-onset pancreatogenic diabetes over the subsequent five years. A chronological summary of key clinical events is provided in Table 1.

**Table 1:** Chronological summary of clinical events. BMI = body mass index; ERCP = endoscopic retrograde cholangiopancreatography; EUS = endoscopic ultrasound; HbA1c = glycated hemoglobin; MPD = main pancreatic duct; MRCP = magnetic resonance cholangiopancreatography; PERT = pancreatic enzyme replacement therapy; PTC = percutaneous transhepatic cholangioplasty

Time point	Clinical event
Year 0	Deceased donor orthotopic liver transplantation; deceased donor kidney transplantation performed two days later
Year 0 (postoperative)	Biliary anastomotic stricture identified; ERCP attempted
Year 0 (postoperative)	ERCP complicated by biliary perforation and bile leak
Year 0 (postoperative)	PTC with serial cholangioplasty; complete biliary stricture resolution achieved
Years 0–5	Donor-derived hepatitis C virus treated and virologically cured; stable dual allograft function maintained
Year 5	Presentation with chronic epigastric pain, 30-pound unintentional weight loss, severe cachexia (BMI: 16.2 kg/m²)
Year 5	MRCP: diffuse MPD dilation with intraductal filling defects up to 7 mm; EUS: severe chronic pancreatitis, parenchymal atrophy, intraductal calculi up to 8 mm in a 10 mm dilated MPD
Year 5	Exocrine pancreatic insufficiency confirmed (fecal elastase); PERT initiated. Type 3c diabetes identified (HbA1c: 9.6%); insulin pump therapy and continuous glucose monitoring initiated
Year 5	ERCP #1: patent biliary sphincterotomy; deep pancreatic duct cannulation unsuccessful due to inflammatory distal bile duct stricture distorting ampullary anatomy
Year 5 + 10 weeks	ERCP #2: successful deep pancreatic duct cannulation (0.035-inch hydrophilic guidewire); sphincterotomy extended to 5 mm; 4 mm balloon dilation of pancreatic head stenosis; debris extraction; 5 Fr × 12 cm plastic stent placed
Year 5 + 20 weeks	ERCP #3: stent exchange performed; ductal patency confirmed
Year 5 + 30 weeks	ERCP #4: stent upsized to 7 Fr × 12 cm plastic stent for ductal remodeling
Year 5 + 9 months	ERCP #5: stent removed electively; no procedural complications. Patient demonstrating weight gain, reduced pain frequency, improved oral intake, and improved glycemic control

At the current presentation, physical examination revealed severe cachexia with a body mass index (BMI) of 16.2 kg/m². Notably, her weight had remained stable at approximately 125-130 pounds during her routine five-year post-transplant follow-up, before rapidly declining to 90 pounds over the preceding nine months.

**Figure 1:**
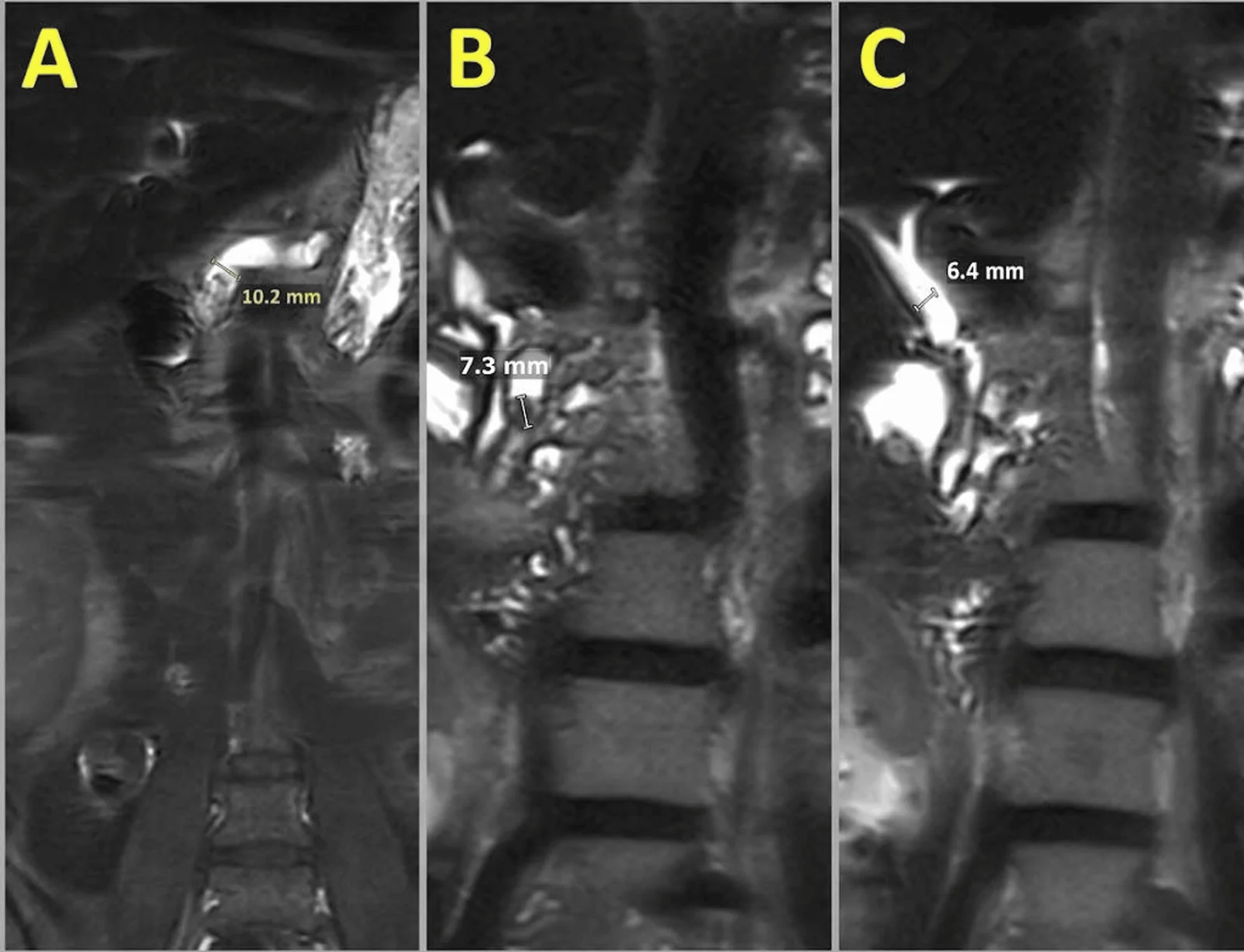
Magnetic resonance cholangiopancreatography (coronal T2-weighted). (A) Dilatation of the main pancreatic duct (MPD). (B) An MPD stone. (C) Dilatation of the common bile duct.

**Figure 2:**
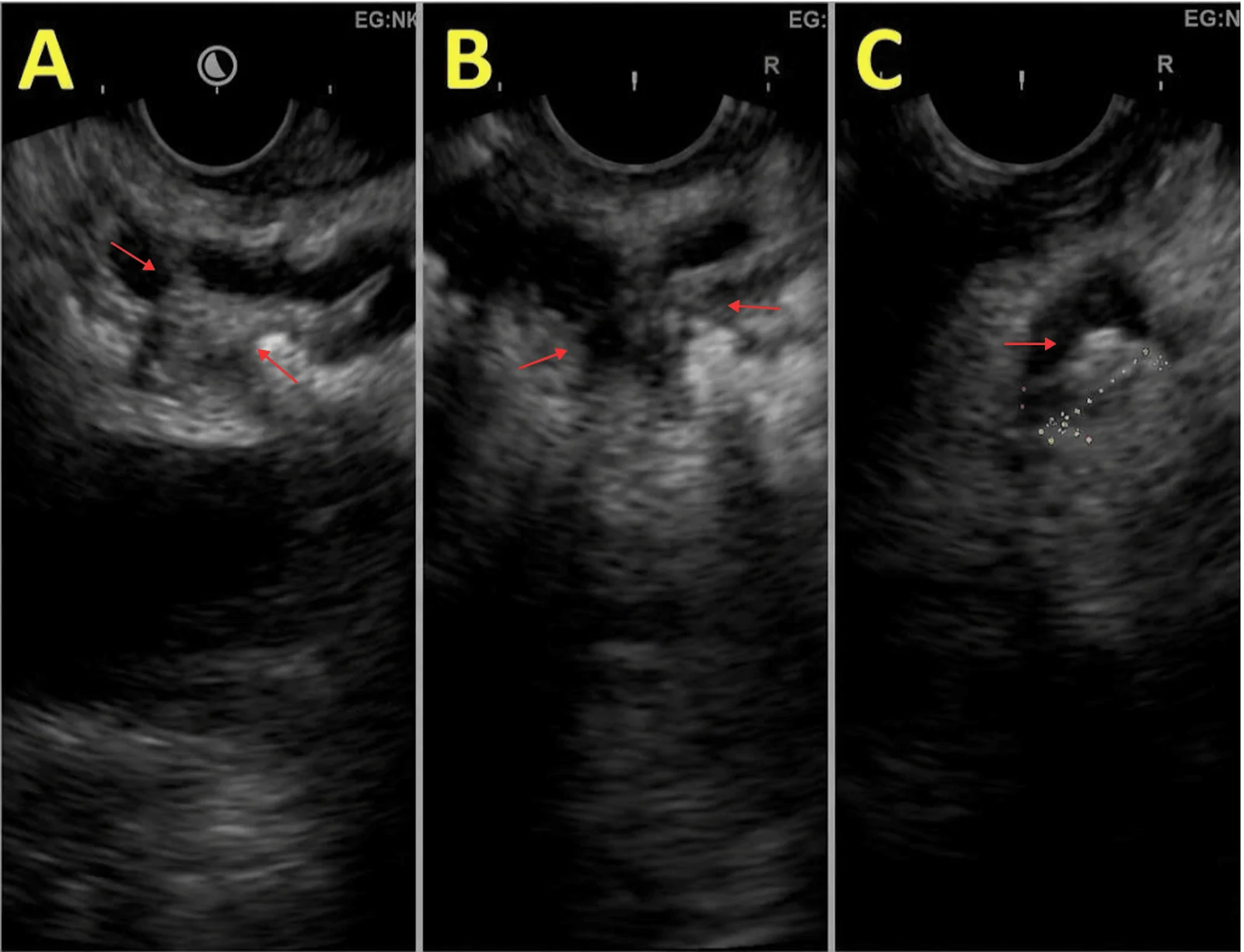
Endoscopic ultrasound findings consistent with chronic pancreatitis. (A) The uncinate process demonstrates a relatively hypoechoic and lobular echotexture typical of the ventral anlage. (B) Evaluation of the pancreatic head reveals heterogeneous parenchyma. (C) View of the main pancreatic duct shows prominent ductal dilation containing a distinct intraductal hyperechoic focus with posterior acoustic shadowing, diagnostic of a pancreatic calculus.

The patient reported bloating, early satiety, and difficulty tolerating solid foods. Complete blood count and comprehensive metabolic panel were within normal limits, with stable renal and hepatic allograft function. Fecal elastase was markedly reduced, confirming exocrine pancreatic insufficiency, and pancreatic enzyme replacement therapy (PERT) was initiated. Glycated hemoglobin (HbA1c) was elevated at 9.6%, consistent with type 3c (pancreatogenic) diabetes in the setting of diffuse pancreatic fibrosis, corticosteroid-induced insulin resistance, and tacrolimus-mediated β-cell toxicity. Importantly, clinical records indicate that routine therapeutic drug monitoring was performed throughout her clinical course, with no documented supratherapeutic tacrolimus trough levels to suggest acute drug toxicity as the primary trigger. Continuous glucose monitoring and subcutaneous insulin pump therapy were initiated in collaboration with endocrinology. Magnetic resonance cholangiopancreatography (MRCP) demonstrated diffuse main pancreatic duct (MPD) dilation (Figure 1A) with intraductal filling defects up to 7 mm consistent with ductal calculi (Figure 1B), along with associated dilation of the common bile duct (Figure 1C). Endoscopic ultrasound (EUS) confirmed severe chronic pancreatitis with hypoechoic, lobular parenchyma involving the uncinate process (Figure 2A) and heterogeneous echotexture of the pancreatic head (Figure 2B), along with intraductal calculi up to 8 mm within a 10 mm dilated MPD, visualized as a hyperechoic focus with posterior acoustic shadowing (Figure 2C). The small discrepancy in stone size between modalities is attributable to differences in spatial resolution between MRCP and EUS, with EUS providing superior stone characterization.

**Figure 3:**
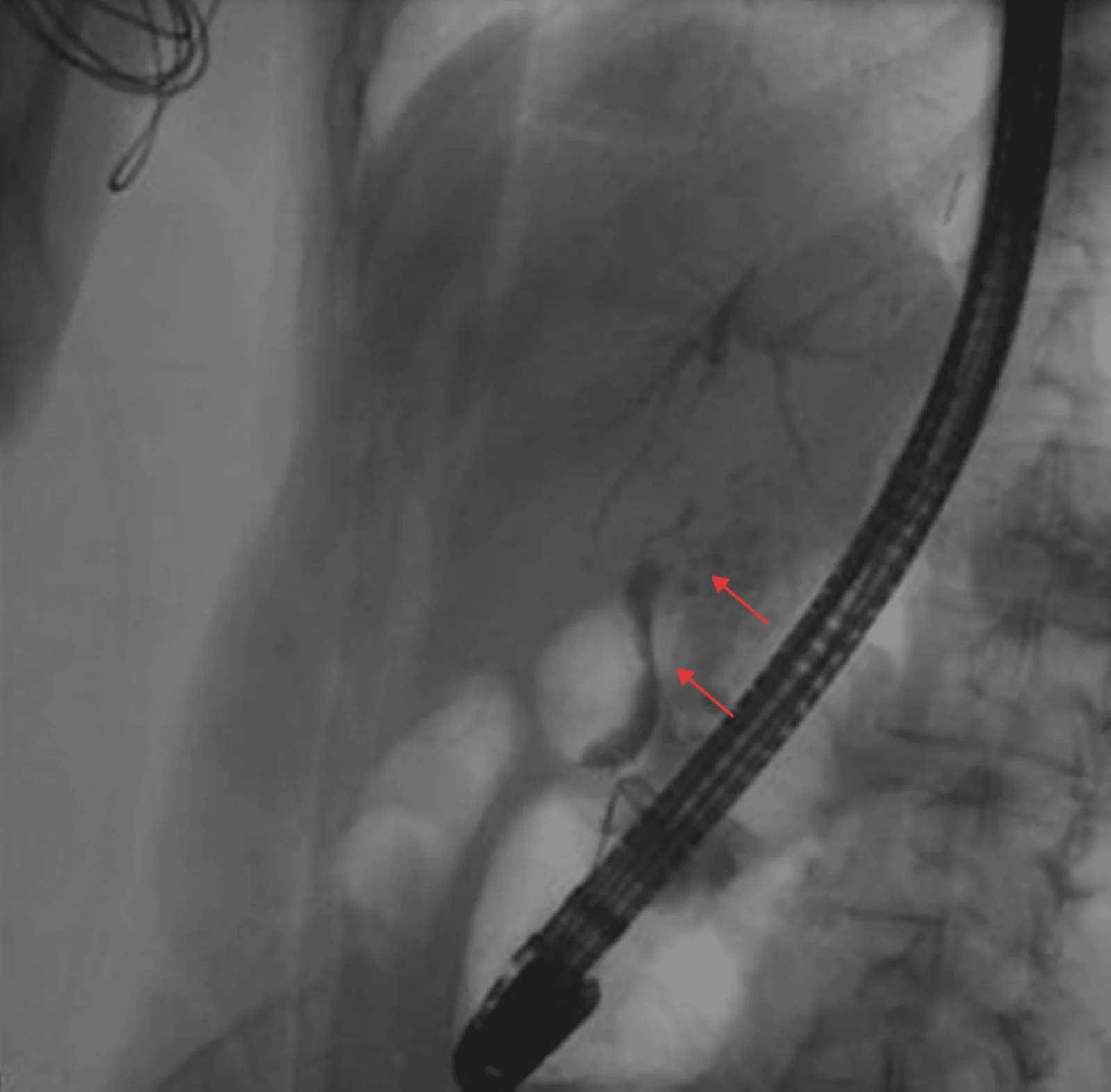
Initial endoscopic retrograde cholangiopancreatography (ERCP). Fluoroscopic image obtained during initial ERCP demonstrating prior biliary sphincterotomy with contrast opacification of the biliary tree. The main pancreatic duct is markedly dilated with multiple intraductal filling defects as indicated by the arrows, consistent with pancreatic duct calculi. Deep pancreatic duct cannulation was unsuccessful because of distal inflammatory distortion of the ampullary region.

**Figure 4:**
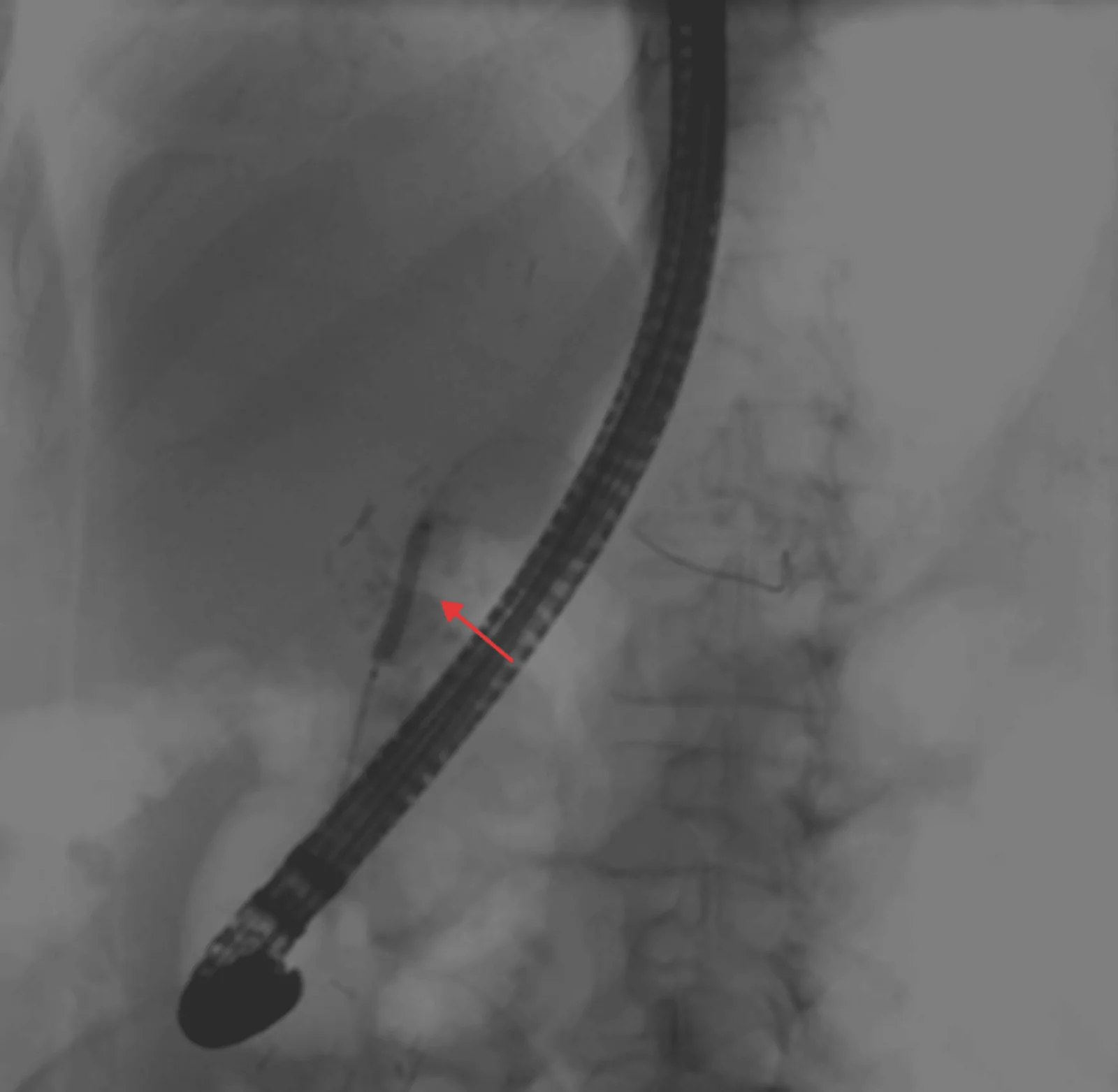
Therapeutic endoscopic retrograde cholangiopancreatography (ERCP) with pancreatic duct stenting. Fluoroscopic image during repeat ERCP demonstrating successful deep cannulation of the ventral pancreatic duct with guidewire access. Contrast injection shows marked dilation of the main pancreatic duct with a dominant stricture in the pancreatic head. Following balloon dilation and ductal clearance, a 5 Fr × 12 cm plastic pancreatic duct stent was placed across the stenosis, indicated by the arrow.

**Figure 5:**
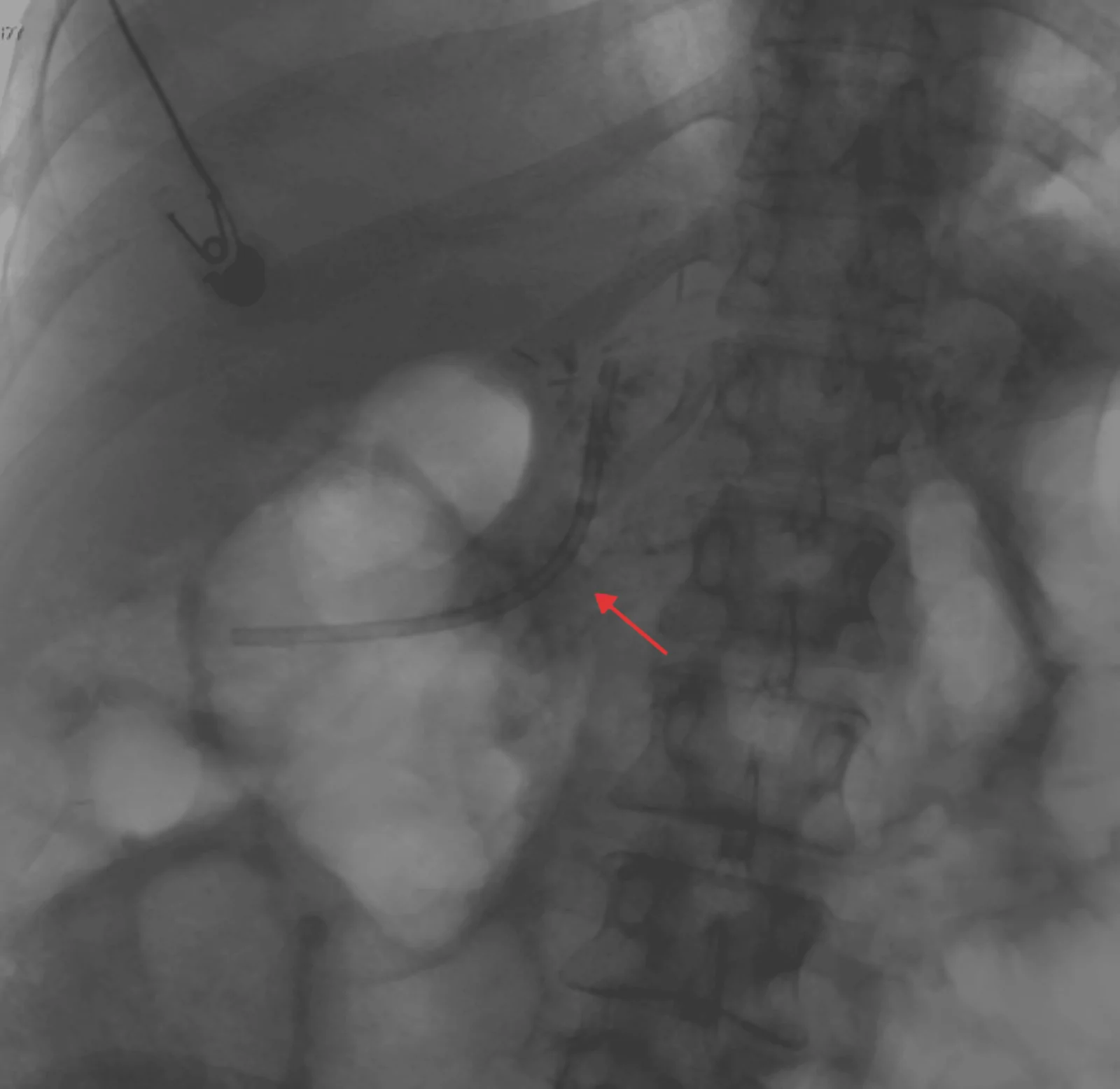
Follow-up endoscopic retrograde cholangiopancreatography (ERCP) with stent exchange. Fluoroscopic image obtained during follow-up ERCP demonstrating exchange of the prior pancreatic duct stent as indicated by the arrow, with maintained ductal patency and improved drainage of the main pancreatic duct.

The initial ERCP, performed at the time of EUS, identified a patent prior biliary sphincterotomy but was unable to achieve deep pancreatic duct cannulation due to an inflammatory stricture of the distal common bile duct distorting ampullary orientation (Figure 3). Given the technical failure, the procedure was terminated, and a repeat ERCP was planned following optimization of the patient’s nutritional status. A subsequent ERCP performed 10 weeks later was successful. The ventral pancreatic sphincterotomy was extended to 5 mm using a pull-type sphincterotome. Deep ductal cannulation was achieved using a 0.035-inch hydrophilic guidewire (Jagwire®, Boston Scientific, Marlborough, MA) after standard wire failure; a 4 mm balloon was used to dilate a severe stenosis in the pancreatic head. The duct was swept with an 8.5 mm extraction balloon, clearing debris, and a 5 Fr × 12 cm plastic stent was placed across the dominant stricture (Figure 4). Serial ERCP procedures were subsequently performed for stent exchange and progressive upsizing to 7 Fr to promote ductal remodeling (Figure 5).

Despite ductal decompression and escalating PERT, intermittent pain and bloating persisted, consistent with the multifactorial nature of chronic pancreatitis pain, including neuropathic and central sensitization components. Lateral pancreaticojejunostomy (Puestow procedure) was discussed in multidisciplinary conference but deferred due to prohibitive operative risk attributable to her dual transplant status, triple immunosuppression, severe cachexia, and labile glycemic control. Nine months after the initial ERCP, the pancreatic stent was electively removed without complications. At the most recent follow-up, the patient showed weight gain (from 90 to 100.4 lbs), better eating habits (tolerating solid foods), and improved blood sugar control (A1c dropped from 9.6% to 7.2%, with daily levels between 70 and 140 mg/dL). She continues active multidisciplinary monitoring across gastroenterology, transplant surgery, endocrinology, and nephrology, with surgical decompression to be reconsidered as her nutritional and metabolic status permits.

## Discussion

This case illustrates the compounded clinical challenges of managing obstructive chronic pancreatitis in a dual solid organ transplant recipient with prior ERCP-related biliary perforation, severe cachexia, and type 3c diabetes, a constellation of findings not previously reported in the literature. The unique complexity of this case lies in the intersection of three independent high-risk domains: post-transplant immunosuppression, prior altered biliopancreatic anatomy, and advanced exocrine-endocrine failure.

The biliary complications in this case highlight an infrequently reported but well-recognized risk of ERCP-related perforation in the post-OLT setting. Localized inflammation, ischemia, and anastomotic fibrosis render the ductal wall susceptible to instrumentation injury, even during technically routine procedures [9]. Emergent PTC with serial cholangioplasty achieved complete stricture resolution in this patient, supporting PTC as an effective rescue strategy when endoscopic management fails or is complicated [3].

Pancreatic ductal access was technically demanding in this case for two compounding reasons: the pre-existing biliary sphincterotomy altered ampullary orientation, and the inflammatory distal bile duct stricture obscured the pancreatic orifice, contributing to failed cannulation at the initial ERCP. At the subsequent procedure, a 0.035-inch hydrophilic guidewire enabled deep ductal access after standard wire failure, a technique consistent with established guidance for difficult pancreatic cannulation in post-transplant anatomy [10,11].

The landmark randomized controlled trial by Cahen et al. first demonstrated surgical superiority over endoscopy for painful obstructive chronic pancreatitis, with pain relief achieved in 75% of surgical versus 32% of endoscopic patients at 24 months [12]. The subsequent ESCAPE trial corroborated these findings (58% vs. 39% pain relief at 18 months) and demonstrated greater cost-effectiveness with early surgery [13]. However, both trials enrolled patients who were candidates for either intervention. The present patient with dual transplant status, triple immunosuppression, severe cachexia, and brittle type 3c diabetes was not a surgical candidate, and these data cannot be directly extrapolated. Notably, ESCAPE subgroup analysis suggested comparable pain reduction when complete endoscopic ductal clearance was achieved, supporting a targeted endoscopic strategy in appropriately selected high-risk patients.

Current guidelines recommend ESWL for pancreatic duct stones greater than 5 mm that are not extractable by conventional ERCP [11,14]. Given calculi up to 8 mm, ESWL would have been a guideline-concordant and reasonable option in this patient but was unavailable at the treating institution. Pancreatoscopy-directed electrohydraulic or laser lithotripsy is an emerging alternative with similarly limited availability at many centers [11]. Given the unavailability of ESWL, we focused on what endoscopy could realistically achieve: reducing ductal pressure and gradually remodeling the dominant stricture through sequential stent upsizing from 5 Fr to 7 Fr. Pain improved, but only partially, which is not surprising. Ductal decompression addresses obstructive physiology, but chronic pancreatitis pain has a life of its own, driven by central sensitization and neuropathic rewiring that no stent can reach [15,16].

Her diabetes added another layer of complexity that deserves its own discussion. Type 3c diabetes does not behave like type 1 or type 2: the concurrent loss of glucagon-secreting alpha cells creates an unpredictable hypoglycemia risk that catches clinicians off guard [17,18]. In this patient, that baseline vulnerability was amplified by two elements of her immunosuppressive regimen: corticosteroid-driven insulin resistance and tacrolimus-mediated β-cell toxicity. Tacrolimus inhibits the calcineurin-NFAT signaling pathway required for insulin gene transcription in β cells and promotes β-cell apoptosis, thereby reducing insulin secretion [7]. The international PTDM consensus guidelines recognize this mechanism, identifying tacrolimus-based immunosuppression as a major modifiable risk factor. However they advise against routinely changing immunosuppression to reduce PTDM risk alone. Adjustment is instead reserved for patients where the burden of diabetes outweighs the risk of rejection [8]. Standard insulin regimens were insufficient; stabilization ultimately required continuous subcutaneous insulin infusion with real-time glucose monitoring, a level of glycemic management that is difficult to achieve without dedicated endocrinology partnership [18]. Exocrine insufficiency driving malabsorption and cachexia was managed with escalating PERT per current evidence-based guidelines [19].

## Conclusions

This case demonstrates that serial ERCP with progressive pancreatic duct stenting is a feasible, personalized approach for managing obstructive chronic pancreatitis in carefully selected high-risk dual solid organ transplant recipients when surgical decompression carries prohibitive risk. Glycemic management in these patients is rarely straightforward, and the overlapping effects of tacrolimus, corticosteroids, and progressive pancreatic fibrosis demand early and ongoing endocrinology input rather than a reactive approach. Beyond diabetes, the multiple organ systems involved make isolated management insufficient, with such patients requiring skilled and collaborative multidisciplinary teams. Cases like this highlight a broader gap in the literature. Further studies, including multicenter data, are warranted to define optimal endoscopic and metabolic management strategies in this high-risk population.
